# Children with cancer and their cardiorespiratory fitness and physical function—the long-term effects of a physical activity program during treatment: a multicenter non-randomized controlled trial

**DOI:** 10.1007/s11764-023-01499-7

**Published:** 2023-12-06

**Authors:** Martin Kaj Fridh, Peter Schmidt-Andersen, Liv Andrés-Jensen, Troels Thorsteinsson, Peder Skov Wehner, Henrik Hasle, Kjeld Schmiegelow, Hanne Bækgaard Larsen

**Affiliations:** 1 Department of Pediatrics and Adolescent Medicine, Copenhagen University Hospital - Rigshospitalet, Copenhagen, Denmark; 2 The University of Copenhagen, Faculty of Health Science, Institute for Clinical Medicine, Copenhagen, Denmark; 3 Department of Occupational Therapy and Physiotherapy, Centre of Head and Orthopedics, Copenhagen University Hospital - Rigshospitalet, Copenhagen, Denmark; 4 Department of Pediatric Hematology and Oncology, Hans Christian Andersen Children’s Hospital, Odense University Hospital, Odense, Denmark; 5 Department of Pediatrics and Adolescent Medicine, Aarhus University Hospital, Aarhus, Denmark; 6 Faculty of Health and Medical Sciences, University of Copenhagen, Copenhagen, Denmark

**Keywords:** Childhood cancer, Cardiorespiratory fitness, Muscle strength, Physical activity intervention, Peer support intervention

## Abstract

**Purpose:**

We aimed to determine the effects of a classmate-supported, supervised, in-hospital physical activity program during treatment primarily on cardiorespiratory fitness and secondarily on physical function.

**Methods:**

A multicenter non-randomized controlled intervention study including children diagnosed with cancer, 6–18 years at diagnosis treated with chemo-/radiotherapy. The intervention comprised (i) an educational session on cancer in the child’s school class; (ii) selection of two “ambassadors”—classmates who were co-admitted, supporting the child’s everyday hospital life; and (iii) supervised in-hospital physical activity from diagnosis and throughout intensive treatment. One-year post-treatment, physical testing included cardiorespiratory fitness (primary outcome), Sit-to-Stand test, Timed-Up-and-Go, and Handgrip Strength.

**Results:**

The intervention group included 75 of 120 children (61% boys, 13.4 ± 3.1 years); the control groups included 33 of 58 children with cancer (58% boys, 13.5 ± 2.5 years), and 94 age- and sex-matched children without a cancer history. One-year post-treatment, cardiorespiratory fitness tended to be higher in the intervention group (37.0 ± 6.0 mL/kg/min) than in the patient control group with cancer (32.3 ± 9.7 mL/kg/min) (mean difference 4.7 [0.4 to 9.1], *p* = 0.034). The intervention group performed better in the secondary outcomes. Compared with community controls, both patient groups had lower cardiorespiratory fitness. The patient control group had lower Sit-to-Stand, Timed Up and Go, and Handgrip Strength, while the intervention group had strength comparable to that of the community controls.

**Conclusions:**

Peer-supported, supervised, in-hospital physical activity during treatment may improve cardiorespiratory fitness and muscle strength 1-year post-treatment in children with cancer; however, survivors continue to have lower cardiorespiratory fitness than community controls.

**Implications for Cancer Survivors:**

Children with cancer may benefit from in-hospital physical activity in improving long-term cardiorespiratory fitness and muscle strength.

**Supplementary Information:**

The online version contains supplementary material available at 10.1007/s11764-023-01499-7.

## Background

The improvements in childhood cancer survival rates create a need to lessen long-term treatment-related late effects to promote the best possible return to everyday life, including social, academic, and physical activities [1, 2]. Childhood survivors of cancer (CCS) experience prolonged absence from school, sports, and leisure activities during treatment, reducing their peer interaction and disrupting their development of social skills [3–5]. Impaired cardiorespiratory fitness [6–10], muscle strength [11, 12], and physical performance [11, 13] are common long-term physiological consequences of anti-cancer treatment, affecting CCS’ ability to perform activities of daily living and their self-perception [14, 15] and reducing their ability to fully participate in social activities and education [16, 17]. Consequently, the CCS are vulnerable to social exclusion [3], which further diminishes their incentive to be physically active [18, 19]. Taken together, the impairments in physical and social functioning impact their health-related quality of life [20, 21]. Accordingly, there is an urgent call for interventions to address these aspects to ensure the children’s optimal return to everyday life after treatment.

At diagnosis, we initiated a multimodal intervention entitled “Rehabilitation including Social and Physical Activity and Education in Children and Teenagers with Cancer” (RESPECT), which included hospital “co-admission” of healthy classmates as *ambassadors* to support the children with cancer and to promote the social network between hospital, school, and peers. This was combined with a supervised in-hospital physical activity program [22, 23]. The intervention was initiated at diagnosis to maintain both social relationships and physical functioning because ambassadors can increase the motivation of the child with cancer to engage in physical activity [24–26]. The overall aim of the RESPECT study is to facilitate children with cancer’s reentry into everyday life after treatment, including adequate physical performance. We have previously shown that this intervention can maintain the children’s cardiorespiratory fitness and physical function during the first six months of treatment, whereas children receiving usual care experienced a decline in cardiorespiratory fitness and physical function [27]. Therefore, the primary objective of the present study was to investigate the effects of a multimodal social and physical activity intervention on cardiorespiratory fitness, muscle strength, and physical function 1-year post-treatment when compared with both CCS controls and children not previously diagnosed with cancer.

## Methods

### Design and setting

This study is a multicenter, prospective, non-randomized controlled multicomponent study entitled “Rehabilitation including Social and Physical Activity and Education in Children and Teenagers with Cancer” (RESPECT) (Clinical Trial registration NCT01772849 and NCT01772862) and is part of the work of the Center for Integrated Rehabilitation (CIRE) [28].

### Participants

**Fig. 1:**
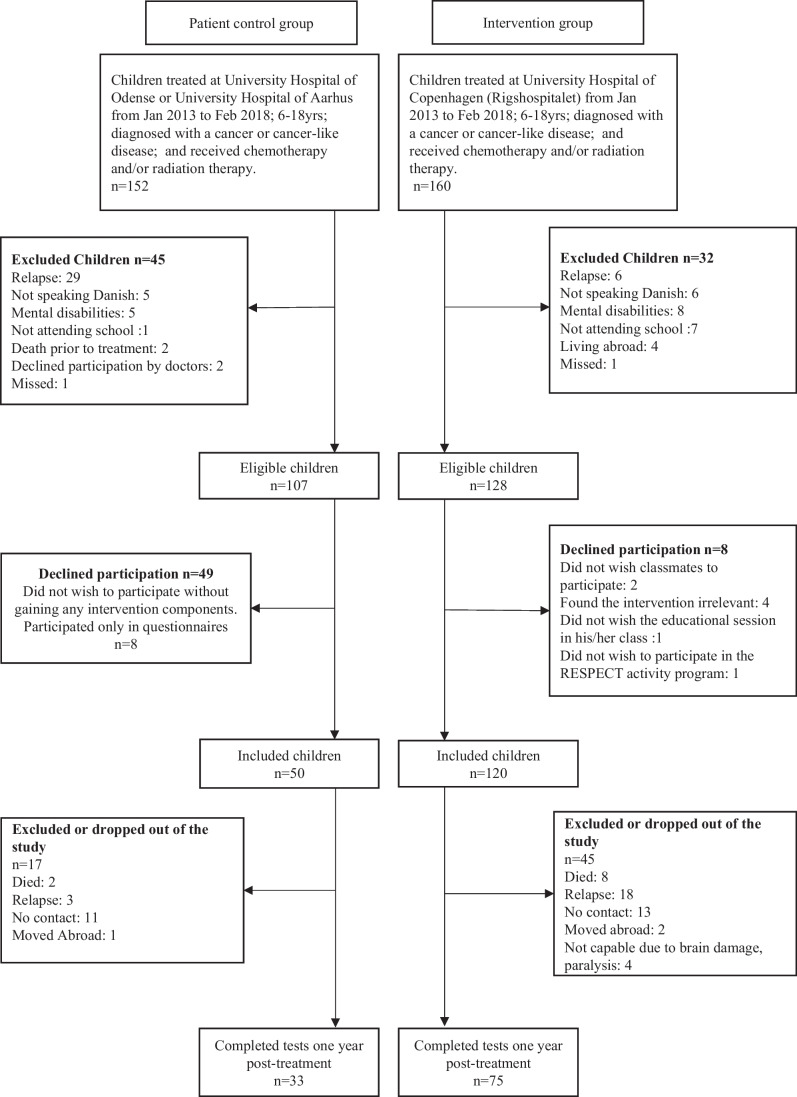
Flowchart of the enrollment process and reasons for dropouts in the RESPECT (Rehabilitation including Social and Physical Activity and Education in Children and Teenagers with Cancer) study

We included participants during January 2013–February 2018. Inclusion criteria were age 6–18 years; any cancer diagnosis or myelodysplastic syndrome (MDS) or Langerhans cell histiocytosis (LCH); treatment with chemotherapy and/or radiation therapy; enrolled in school at diagnosis; and able to communicate in Danish. Exclusion criteria were mental disability (e.g., Down syndrome) and severe co-morbidity. We included children treated at the University Hospital of Copenhagen, Rigshospitalet, in the intervention group, and children treated at Odense University Hospital and Aarhus University Hospital in the patient control group. The patient control group received standard institutional, guided care. We excluded participants if they had experienced a recurrence of their primary diagnosis or were diagnosed with a secondary cancer. Further, we included age- and sex-matched children without a cancer history and/or chemotherapy/radiation as a community control group. The community control group consisted of a subgroup of ambassadors (*n* = 64) and a subgroup of participants without a history of cancer from the Acute Lymphoblastic Leukemia Survivor Toxicity and Rehabilitation (ALL-STAR) study (*n* = 30) assessed at the University Hospital of Copenhagen [29]. Figure 1 shows the enrollment process.

### Intervention components

The intervention consisted of three components. (1) We conducted a 90-min educational session for the child with cancer’s school class on cancer treatment and its side effects, everyday life at the hospital, supportive care, the benefits of physical activity, and the RESPECT study. (2) We selected two classmates as “*ambassadors*” in collaboration with the teachers, the classmates’ parents, and the child with cancer [30]. The ambassadors were invited to be co-admitted every 14th in- and outpatient day throughout the entire treatment period. The ambassadors were co-admitted to the hospital for the day (i.e., 9 a.m. to 3 p.m.) and were present during the daily routines at the department and participated in school, social, and physical activities. The primary role of the ambassadors was to provide peer support, maintain social inclusion, and increase the motivation of the child with cancer to engage in school and physical activities. The planning of an ambassador co-admission has been presented previously [27]. (3) We conducted an in-hospital supervised physical activity intervention (the RESPECT physical activity program) carried out during admissions to the Department of Pediatric Oncology. The RESPECT physical activity program consisted of individually designed activities (duration 5–30 min) offered three times per week (Monday, Wednesday, and Friday) and group sessions (duration 30–120 min) including all eligible children with cancer and their ambassadors on Tuesdays and Thursdays, as shown in Table 1. Daily, we designed each physical activity session to accommodate the wellbeing (e.g., presence of nausea, pain, and dizziness), training category (able to walk/not in isolation, able to walk/ in isolation, and bedbound), and physical capacity of the child with cancer (Table 1). We had not pre-defined a targeted intensity of the physical activity program before study initiation. The aim of the physical activity sessions was to mobilize the children and accomplish as high an intensity as possible on a given day. Each session started with cardiorespiratory fitness exercises spanning simple mobilization to targeted aerobic exercises (provided the child’s wellbeing permitted) followed by activities and/or games designed to improve muscle strength and balance [31]. Key equipment consisted of stationary cycle-ergometers, treadmills, dumbbells, balls, and various other items to facilitate games. We previously reported the intensity during group sessions elsewhere [6]. We measured the intensity of the individual and group physical activity program in a subgroup of CCS (*n* = 50) from September 2013 to September 2015. The mean heart rate was 145 beats/min [95% CI 142 to 149] or 69.3% [68.1 to 70.4%] of age-specific predicted maximal heart rate. The maximal heart rate was 185 beats/min [95% CI 174 to 184] or 89% [95% CI 87.7% to 90.4%] of age-specific predicted maximal heart rate [6]. Training frequency was calculated by dividing the number of days with physical activity by the number of weekdays admitted to the pediatric oncology department (excluding weekends and holidays).

**Table 1:** The in-hospital RESPECT activity program

Training category/weekday	Monday	Tuesday	Wednesday	Thursday	Friday	Weekend
Able to walk/not in isolation	Individual session 5–30 min Cardiorespiratory fitness Muscle strength Balance	Group session 30–120 min Cardiorespiratory fitness Muscle strength Balance	Individual session 5–30 min Cardiorespiratory fitness Muscle strength Balance	Group session 30–120 min Cardiorespiratory fitness Muscle strength Balance	Individual session 5–30 min Cardiorespiratory fitness Muscle strength Balance	No training
Able to walk/in isolation	Individual session 5–30 min Cardiorespiratory fitness Muscle strength Balance	Individual session 5–30 min Cardiorespiratory fitness Muscle strength Balance	Individual session 5–30 min Cardiorespiratory fitness Muscle strength Balance	Individual session 5–30 min Cardiorespiratory fitness Muscle strength Balance	Individual session 5–30 min Cardiorespiratory fitness Muscle strength Balance	No training
Bedbound	Individual session 5–30 min Muscle strength	Individual session 5–30 min Muscle strength	Individual session 5–30 min Muscle strength	Individual session 5–30 min Muscle strength	Individual session 5–30 min Muscle strength	No training

*RESPECT*, Rehabilitation including Social and Physical Activity and Education in Children and Teenagers with Cancer

### Anthropometry, body composition, and medical characteristics

We weighed the participants to the nearest 0.1 kg and measured height to the nearest 0.1 cm. Body mass index (BMI) was calculated by dividing weight by height^2^.

### Physical outcome evaluation

The primary outcome was VO_2_peak measured with the cardiopulmonary exercise test (CPET). The secondary outcomes were Sit-to-Stand, Timed Up and Go, and Handgrip Strength. We carried out the tests 1-year post-treatment ± 180 days. The treating physician permitted the tests providing the child’s thrombocyte count was > 10 billion/L, hemoglobin count was > 5 mmol/L, and the temperature was < 38°. Exclusion criteria (for testing) included active diarrhea, cough or a cold, and side effects preventing testing. We held annual meetings with all centers to ensure comparability, and we distributed instruction videos to all members of the test teams. The tests are described in detail elsewhere [22]. All children in the age- and sex-matched control group were tested at Copenhagen University Hospital, Rigshospitalet, using the same equipment as the intervention group.

Following a modified Godfrey protocol, we performed the CPET on an electronically braked cycle ergometer (Lode Corival Pediatric or Monark Ergomedic 839 E) [22, 32]. We determined breath-by-breath ventilation and gas exchange data (INNOCOR ergo-spirometry-system, INNO00010, Innovision, DK-5260 Odense, Denmark, or Jaeger Master Screen® CPX System (MS-CPX) and JLAB Software Package™). VO_2_peak was defined as the highest mean over 60 s and expressed in mL/kg/min. The maximal watt of the test was recorded. Heart rate and oxygen saturation were measured every 30 s (Polar FT2 sport tester Polar Electro, Kemple, Finland). Following consultation with experts on CPET testing in healthy children [33], we considered the CPET to be valid if one subjective criterion and two objective criteria were fulfilled. The subjective criteria were signs of intense effort. The objective criteria were heart rate > 180 beats/min and respiratory exchange ratio > 1.05 [33]. We stopped the test if oxygen saturation was under 90 or the child could not maintain the minimum required tempo (70 rpm).

### Physical function tests

The children performed the Sit-to-Stand test [34] using a chair that allowed the child to flex the legs at a 90° angle. The child was instructed to fold his/her arms across the chest or to let them hang to the side, stand straight, and then touch the chair with their bottom while returning to a seated position. Strong verbal encouragement was given during the test. The test score equated the number of repetitions during a 30 second period.

The children performed the Timed Up and Go 3-m test [35] using a chair that allowed the child to flex the legs at a 90° angle. From the start position, with the back resting against the chair and arms on knees, we instructed the child to stand up, walk 3 m as fast as possible, turn around, and return to the start position. Completion time was recorded in seconds to the nearest two decimals. Strong verbal encouragement was given during the test. The last score of three tries was used in the analysis.

Handgrip Strength was measured using a Saehan hand dynamometer (Glanford Electronics, Scunthorpe, UK) and measured in kilograms. Two attempts per arm were performed either standing or sitting and without use of the elbow or the dynamometer touching anything. Strong verbal encouragement was given during the test and the highest score was used in the analysis [36].

### Ethics approval and consent to participate

All participants and their parents gave written informed consent to participate in the educational sessions, to the inclusion of ambassadors, and to participation in the RESPECT activity program. The study was approved by the Regional Ethics Committee for the Capital Region (file. H 3-2012-105) and the Danish Data Protection Agency (file. 2007-58-0015/nr.30-0734) and complies with the Helsinki II Declaration.

### Statistical method

The power calculation is based on the primary endpoint 1-year post-treatment being VO_2_peak, and the power calculation is based on an anticipated 10% higher VO_2_peak in the intervention group compared with the control group. We based the power calculation on a pilot that found a baseline VO_2_peak of 24.3 (SD 5.9) [37]. The significance level 1 year after treatment end was 0.025, and the power was 0.90, resulting in 120 children in each group of children with cancer [22].

We analyzed VO_2_peak (mL/kg/min), VO_2_peak (L/min), max watt, Sit-to-Stand, Timed Up and Go, and Handgrip Strength using analysis of covariance (ANCOVA) models with the residual variance depending on the group (intervention group, patient control group, and community control group). VO_2_peak (L/min)_,_ Sit-to-Stand, Timed Up and Go, and Handgrip Strength were log-transformed before analyses, and the back-transformed relative effects were presented as percentage difference to the reference level. To investigate whether the impact of adjusting for the differences between the three groups could result from differences in sex, age, cancer diagnosis, and time since diagnosis, the groups were compared in three different models: (1) a raw model without any adjustments, (2) a model adjusted for the sex-dependent effects of relative age differences (10% increase in age), and (3) a model further adjusted for cancer-type-dependent effects of time since diagnosis. We categorized the types of cancers in three groups: (1) hematological cancers receiving maintenance therapy (i.e., acute lymphoblastic leukemia (ALL), acute promyelocytic leukemia, t-cell non-Hodgkin’s lymphoma); (2) other hematological cancers (i.e., Hodgkin’s lymphoma, Burkitt non-Hodgkin lymphoma, acute myeloblastic leukemia, myelodysplastic syndrome, Langerhans cell histiocytosis and children with acute lymphoblastic leukemia who were treated with hematopoietic stem cell transplantation); and (3) other oncological diseases (extracranial solid tumors and tumors located in the central nervous system).

We categorized the types of cancers in these three groups based on two previous observations. Firstly, we previously showed that children with acute lymphoblastic leukemia responded differently to physical activity than other oncological diseases (extracranial solid tumors and tumors located in the central nervous system) but not between children with extracranial solid tumors and children with tumors located in the central nervous system [38]. Secondly, we decided to account for the time since the diagnosis, as length of treatment could affect the results. In addition, we evaluated whether the difference between the three groups (intervention group, patient control group, and community control group) depended on sex or age by adding two-factor interactions as well as three-factor interactions between group, sex, and relative age difference to Model 3. The three-factor interaction was insignificant for all outcomes (all *p* > 0.18), and the two-factor interaction between group and sex was insignificant for all outcomes (all *p* > 0.20). However, for some outcomes, the differences between the groups appeared to depend on age. Therefore, estimated group differences for age 8 years and age 18 years are presented for all outcomes. We performed all analyses in R (version 3.6.0) and R-studio.

## Results

### Participant characteristics

We included 120 of 128 (94%) eligible children in the intervention group and 58 of 107 (54%) eligible children in the control group. In the intervention group, two children did not wish classmates to participate, four children found the intervention irrelevant, one child did not wish the educational session in his/her class, and one child did not wish to participate in the RESPECTS activity program. In the patient control group, the children declined participation because they did not gain any intervention components. One-year post-treatment, 45 children had been excluded or had dropped out of the intervention group, and 17 had been excluded or had dropped out of the patient control group (Fig. 1). Thus, the intervention group consists of 75 CCS and the patient control group consisted of 33 CCS at 1-year post-treatment. We observed no difference between groups in age, sex, height, weight, BMI, or diagnosis distribution. Anthropometric and clinical characteristics are shown in Table 2. The treatment protocols of the included children are presented in Supplementary 1.

**Table 2:** Anthropometric and clinical characteristics

Anthropometric characteristics	Intervention group (*n* = 75)	Patient control group (*n* = 33)	*p* value between intervention and patient control	Community control group (*n* = 94)	*p* value between intervention and community control group	*p* value between patient control group and community control group
Sex (males/females)	45/30 (61%/39%)	19/14 (58%/42%)	0.59	55/39 (59%/41%)	0.56	0.95
Age (years)	13.4 ± 3.1	13.5 ± 2.5	0.78	12.9 ±3.0	0.28	0.42
Height (m)	1.58 ± 0.16	1.63 ± 0.16	0.13	1.6 ± 0.16	0.27	0.54
Weight (kg)	51.87 ± 16.18	53.9 ± 15.29	0.54	51.3 ± 16.2	0.82	0.43
BMI (kg/m^2^)	20.4 ± 4.5	19.9 ± 3.3	0.52	19.4 ±3.5	0.10	0.51
Diagnosis			0.69			
Leukemia	37 (49%)	16 (48%)				
Lymphoma	16 (21%)	7 (21%)				
Extracranial solid tumors	16 (21%)	9 (27%)				
Central nervous system tumor	4 (5%)	1 (3%)				
Other hematological disease	3 (4%)	0 (0%)				
Days since diagnosis (median, 10th to 90th percentile)	710 [486 to 1307]	644 [504 to 1314]	0.96			
Physical capacity at diagnosis						
VO_2_peak (mL/min/kg)	27.8 ± 7.2	29.3 ±7.3	0.98			
VO_2_ (mL/min)	1.4 ± 0.5	1.5 ± 0.8	0.25			
Max watt	108 ± 35	119 ± 70	0.17			
Sit-To-Stand (reps)	26 ± 7	18 ± 5	0.01			
Timed-Up-and-Go	3.9 ± 0.8	5.3 ± 1.6	< 0.001			
Right Handgrip Strength (kg)	21 ± 11	18 ± 11	0.19			
Left Handgrip Strength (kg)	19 ± 10	17 ± 12	0.5			

### Training frequency, harms, and feasibility

The median number of physical activity sessions attended per child was 34 [interquartile range: 19 to 50], corresponding to a participation rate of median 64% [interquartile range 50 to 82%] or three sessions per 5 days of in-hospital admission (excluding weekends and holidays). Overall, the children’s participation was spread over a total of 3364 individual and 726 group physical activity sessions. No additional adverse events occurred during the physical activity sessions apart from the six minor events reported in earlier publications: four children experienced minor bruising, one child had a nosebleed during warm-up, and one child fainted shortly after exercise but had no further complications [22, 23].

### Effect of the RESPECT activity program

One-year post-treatment, VO_2_peak tended to be higher in the intervention group compared with the patient control group with a mean difference of 4.7 mL/kg/min [95% CI 0.4 to 9.1 mL/kg/min]. This mean difference remained similar when we adjusted sex-dependent age and for diagnosis-dependent time since diagnosis (Table 3). Watt max and VO_2_ (L/min) during the CPET test were similar between the intervention- and the patient control group. The intervention group had a higher Sit-to-Stand score than that of the patient control group, with a mean difference of 7 repetitions [95% CI 4 to 10]. Moreover, the intervention group completed the Timed Up and Go test faster than the patient control group with a mean difference of − 20% [95% CI − 26 to − 13]. The intervention group was stronger in Handgrip Strength compared with the patient control group in both hands (see Table 3). In Sit-to-Stand, Timed Up and Go, and Handgrip Strength, the mean difference remained similar when we adjusted for sex-dependent age and for diagnosis-dependent time since diagnosis (Table 3). The mean and standard deviations and median and 10th to 90th percentile of cardiorespiratory fitness, muscle strength and physical function are presented in supplementary 2.

**Table 3:** RESPECT activity program: cardiorespiratory fitness, muscle strength, and physical function 1-year post-treatment: comparison of the intervention group with the patient control group

	Unadjusted analysis estimate [95% CI]	*p*	Demographic-adjusted* estimate [95% CI]	*p*	Demographic- and diagnosis-adjusted** estimate [95% CI]	*p*	Comparison at age 8 years in a demographic- and diagnosis-adjusted model with age-dependent difference between groups*** estimate [95% CI]	*p*	Comparison at age 18 years in a demographic- and diagnosis-adjusted model with age-dependent difference between groups*** estimate [95% CI]	*p*
VO_2_peak (mL/kg/min)	4.7 [0.4 to 9.1]	0.034	4.7 [0.5 to 8.8]	0.028	4.3 [0.4 to 8.2]	0.033	9.8 [0.4 to 19.2]	0.042	0.6 [− 6.3 to 7.5]	0.86
VO_2_ (L/min) (% of level among patient controls)	14 [− 11 to 47]	0.30	15 [− 3 to 35]	0.09	12 [− 5 to 33]	0.16	27 [− 15 to 90]	0.24	3 [− 23 to 39]	0.82
Watt max (W)	9 [− 20 to 37]	0.55	7 [− 11 to 26]	0.43	3 [− 16 to 21]	0.77	7 [− 37 to 52]	0.74	1 [− 32 to 33]	0.97
Sit-to-Stand (repetitions)	67 [4 to 10]	< 0.001	7 [4 to 10]	< 0.001	7 [4 to 10]	< 0.001	− 1 [− 8 to 6]	0.78	12 [7 to 17]	< 0.001
Timed Up and Go (% of level among patient controls)	− 20 [− 26 to − 13]	< 0.001	− 21 [− 27 to − 14]	< 0.001	− 21 [− 28 to − 14]	< 0.001	− 7 [− 25 to 16]	0.50	− 29 [− 39 to − 18]	< 0.001
Right Handgrip Strength (% of level among patient controls)	24 [− 4 to 61]	0.095	29 [12 to 49]	0.001	31 [12 to 53]	0.001	35 [− 12 to 106]	0.15	29 [− 3 to 70]	0.074
Left Handgrip Strength (% of level among patient controls)	25 [− 1 to 59]	0.065	31 [15 to 48]	< 0.001	32 [16 to 52]	< 0.001	24 [− 14 to 77]	0.23	38 [8 to 77]	0.012

*CI*, confidence interval

*Adjusted for sex-dependent associations with relative age

**Adjusted for sex-dependent associations with relative age and diagnosis-dependent time since diagnosis

***Estimated in a model including sex-dependent associations with relative age, diagnosis-dependent time since diagnosis, and group-dependent associations with relative age

VO_2_ (L/min), Timed Up and Go, Right Handgrip Strength, and Left Handgrip Strength were log-transformed; results are therefore presented as % difference from the level in the patient control group

### Cardiorespiratory fitness, muscle strength, and physical function compared with age- and sex-matched community control group

One-year post-treatment, both the intervention- and patient control group had lower cardiorespiratory fitness than the community control group (mean difference − 4.7 [95% CI − 7.1 to − 4.7]) (Table 4). The intervention group and community control group performed similarly in Sit-to-Stand (mean difference 0 repetitions [95% CI − 2 to 2]), Timed Up and Go (mean difference − 3% [95% CI − 7 to 1]), and Handgrip Strength (right hand: mean difference − 4% [95% CI − 17 to 10], left hand: mean difference − 5% [95% CI − 18 to 10]). However, the patient control group had a lower Sit-to-Stand score (mean difference − 6.29 [− 9.29 to -3.27] repetitions), was slower to complete the Timed Up and Go test (mean 21 [12 to 32] %) and had lower Handgrip Strength (right hand: mean difference − 23 [− 41 to − 1] %, left hand: − 24 [− 40 to − 4] %) than did the community control group (Table 4). The mean differences remained similar when we adjust for sex-dependent age (Table 4).

**Table 4:** RESPECT activity program: cardiorespiratory fitness, muscle strength, and physical function 1-year post-treatment: comparison of the intervention group and the patient control group with the community control group

	Unadjusted analysis estimate [95% CI]	*p*	Demographic-adjusted* estimate [95% CI]	*p*	Estimated value at age 8 years in a model with age-dependent difference between groups** estimate [95% CI]	*p*	Estimated value at age 18 years in a model with age-dependent difference between groups** estimate [95% CI]	*p*
VO_2_ peak (mL/kg/min)								
Intervention group	− 4.7 [− 7.1 to − 4.7]	< 0.001	− 5.4 [− 8.0 to − 2.8]	< 0.001	− 6.7 [− 14.3 to 0.9]	0.081	− 5.0 [− 9.2 to − 0.8]	0.019
Patient control group	− 9.3 [− 13.8 to − 4.7]	< 0.001	− 10.1 [− 14.4 to − 5.7]	< 0.001	− 18.2 [− 29.3 to − 7.2]	0.002	5.1 [− 12.5 to 2.3]	0.17
VO_2_ (L/min) (% of level among Community controls)								
Intervention group	− 16 [− 26 to − 3]	0.016	− 10 [− 17 to − 3]	0.008	− 16 [− 32 to 5]	0.12	− 8 [− 19 to 5]	0.21
Patient control group	− 26 [− 42 to − 5]	0.021	− 22 [− 33 to − 8]	0.004	− 38 [− 58 to − 8]	0.02	− 10 [− 32 to 19]	0.45
Max watt (W)								
Intervention group	− 48 [− 73 to − 23]	< 0.001	− 39 [− 55 to − 23]	< 0.001	9 [− 35 to 53]	0.68	− 61 [− 86 to − 37]	< 0.001
Patient control group	− 56 [− 88 to − 24]	0.001	− 46 [− 66 to − 27]	< 0.001	− 10 [− 61 to 42]	0.70	− 62 [− 95 to − 29]	< 0.001
Sit-to-Stand (repetitions)								
Intervention group	0 [ − 2 to 2]	0.78	0 [− 2 to 2]	0.87	− 4 [− 7 to 0]	0.058	3 [0 to 6]	0.058
Patient control group	− 6 [− 9 to − 3]	< 0.001	− 6 [− 9 to − 3]	< 0.001	− 4 [− 12 to 3]	0.26	− 8 [− 13 to − 2]	0.007
Timed Up and Go (% of level among community controls)								
Intervention group	− 3 [− 7 to 1]	0.14	− 4 [− 9 to 1]	0.083	8 [− 1 to 18]	0.083	− 12 [− 18 to − 5]	< 0.001
Patient control group	21 [12 to 32]	< 0.001	22 [12 to 32]	< 0.001	17 [− 5 to 46]	0.14	23 [6 to 43]	0.009
Right Handgrip Strength (% of level among Community controls)								
Intervention group	− 4 [− 17 to 10]	0.53	− 4 [− 11 to 4]	0.31	− 4 [− 19 to 13]	0.60	− 4 [− 16 to 10]	0.59
Patient control group	− 23 [− 41 to − 1]	0.043	− 26 [− 35 to − 14]	< 0.001	− 30 [− 53 to 3]	0.068	− 23 [− 40 to 0]	0.051
Left Handgrip Strength (% of level among Community controls)								
Intervention group	− 5 [− 18 to 10]	0.49	− 5 [− 12 to 3]	0.24	− 8 [− 23 to 9]	0.32	− 2 [− 14 to 12]	0.78
Patient control group	− 24 [− 40 to − 4]	0.022	− 27 [− 35 to − 18]	< 0.001	− 25 [− 45 to 2]	0.061	− 28 [− 41 to − 11]	0.004

*CI*, confidence interval

*Adjusted for sex-dependent associations with relative age

**Estimated in a model including, sex-dependent associations with relative age, and group-dependent associations with relative age

VO_2_ (L/min), Timed Up and Go, Right Handgrip Strength and Left Handgrip Strength were log-transformed; results are therefore presented as % difference from the level in the community control group

## Discussion

In this multicenter, prospective, non-randomized, controlled, multimodal study, we showed that children who received a peer-supported, supervised in-hospital physical activity program during treatment had higher cardiorespiratory fitness, muscle strength, and physical function than children who received usual care 1-year after ended treatment. Moreover, we showed that children with cancer in the intervention group had similar muscle strength and physical function to children with no history of cancer (i.e., community controls); however, the children with cancer still displayed lower cardiorespiratory fitness than community controls. Our previous study showed that the intervention and patient control groups were comparable in anthropometric and diagnosis distribution prior to inclusion [27]. Our study suggested that children in the intervention group could maintain their cardiorespiratory fitness, muscle strength, and physical function during treatment. In contrast, the control group experienced a further decline in cardiorespiratory fitness [27]. Furthermore, the intervention group and patient control group were comparable in cardiorespiratory fitness and handgrip strength but not in Sit-To-Stand and Timed-Up-and-Go at baseline (Table 2). Collectively, this suggests that a peer-supported, supervised in-hospital physical activity program during treatment may have long-lasting benefits for CCS regarding cardiorespiratory fitness, muscle strength, and physical function. However, the observed differences between the intervention- and patient control groups in physical function may be due to baseline differences. This indicate that children with cancer could benefit from early in-hospital physical activity programs, also in their everyday life after treatment.

It is possible that the improved physical function supported the children’s educational and social rehabilitation as they may have fewer difficulties in matching the physical functioning of peers [39]. These results suggest that physically active children during treatment require less rehabilitation post-treatment to regain age-matched physical function but still require targeted interventions to improve their cardiorespiratory fitness. Conflicting evidence on the effects of physical activity during treatment exists. Several studies show benefits for cardiorespiratory fitness [23, 40–42], muscle strength [43–45], and physical function [46, 47], whereas others show no effect [48, 49]. Collectively, data have been synthesized in two meta-analyses, showing that physical activity during treatment can improve muscle strength [50] and physical function [51]. In agreement with the present study, CCS have lower cardiorespiratory fitness and muscle strength several years post-treatment [8, 9, 11, 52]. Factors that can contribute to lower cardiorespiratory fitness and muscle strength include cardiac, pulmonary, and vascular limitations, as well as peripheral neuropathy and altered body composition [9, 52]. The present study showed that physical activity during treatment has both an immediate effect and a long-term effect manifesting a year after intervention end. The effects of the intervention might reduce the children’s risk of developing cardiorespiratory fitness-related medical conditions for years after their treatment has ended. In adults, studies have shown that a change in VO_2_peak of 1 mL/kg/min corresponds to a 9–10% reduction in the incidence of cardiac mortality [53, 54] and a 5% cardiovascular disease risk reduction [55]. This is further supported by a recent study showing that exercise during childhood cancer treatment maintained left ventricular function post-treatment, whereas this was not the case in a control group with no exercise [56].

The RESPECT project is the first to include healthy classmates as ambassadors during cancer treatment and in a physical activity program [22]. Through semi-structured interviews, we previously explored children with cancer’s motivation to engage in physical activity while admitted to the hospital [57, 58]. The children with cancer described how their motivation to be physically active increased during treatment because their ambassadors participated in the physical activity sessions [57, 58]: the ambassadors’ presence provided distractions from common side effects (i.e., nausea, pain) and everyday hospital life, motivating them to get out of bed. Qualitatively, parents and children have described how the intervention supported the children in re-entering everyday life post-treatment, including physical activities, social interactions, and school attendance [57, 58]. The ambassadors provided an opportunity to receive support from peers when performing physical activities [57]. Moreover, they represented a unique opportunity to incorporate the child’s everyday life into the hospital setting and increase the child’s willingness to engage in rehabilitation offers [57]. However, involving healthy classmates as ambassadors may be more difficult in other settings. Thus, exploring alternative approaches to including healthy children in physical activity programs for children with cancer is critical. Through semi-structured interviews, we previously investigated the experience of being part of the RESPECT study [58]. The parents described how participating in the RESPECT intervention increased their understanding of how anti-cancer treatment and sedentary behavior affected their child’s physical capacity [58]. They expressed that they learnt the importance of physical activity both during and after treatment and that this enabled them to support their child’s physical activity post-treatment [58]. Throughout the study, one exercise professional or physical therapist conducted the physical activity program at a given time. Thus, achieving the physical activity program in other settings requires few additional human resources.

Taken together, the findings show that children with cancer need physical rehabilitation. Without physical rehabilitation the children risk long-term impairments in cardiorespiratory fitness, muscle strength, and physical function. Further, this study indicates that physical activity is beneficial for children with cancer, thus supporting the recommendations from the international Pediatric Oncology Exercise Guidelines (iPOEG) stating that children with cancer should be physically active and do what they can, when they can [59]. Building on the iPOEG guidelines, we recommend that clinicians emphasize physical activity during treatment, when side effects (i.e., nausea, pain) are most common. Targeted exercise interventions including cardiorespiratory fitness may be more suitable later in the treatment trajectory when treatment is less intense (e.g., maintenance phase of ALL treatment) or after treatment end. This remains to be investigated.

### Strengths and limitations

The strength of this study is the high inclusion rate in the intervention group, with 94% of eligible children completing the intervention. Prior to the initiation of the RESPECT study, we expected some selection bias, given the study design. However, the limited participation rate in the control group (47%) introduced the possibility of further selection bias. The number of dropouts and excluded patients at 1-year post-treatment is a limitation of the study. We, therefore, suspected attrition bias because of poor retention one-year post-treatment. Thus, we performed post hoc analyses and tested for systematic dropouts or excluded patients concerning diagnosis distribution, sex, age, socioeconomic status, and ethnicity. Further, we tested whether these variables were comparable at baseline between the intervention and patient control groups. None of these variables were associated with dropout or exclusion in this study. No differences were observed in any of these variables at baseline or one-year post treatment. We observed more relapse cases in the intervention group compared to the patient control group. We expected this as the treatment of most rare and high-risk cancers is centralized at The University Hospital of Copenhagen. This discrepancy in eligible patients between the centers, combined with the high number of non-responders in the control group, limits the generalizability and certainty of the study results.

There is a possible geographical difference between The University Hospital of Copenhagen and the rest of the country concerning the testing personnel and differences in standard institutional guided care unrelated to treatment protocols. Nevertheless, all institutions are subject to the same regulations and have the same financial resources available to treat children. We included all children who received chemotherapy and/or radiation therapy. Consequently, the study consists of a heterogeneous group; therefore, we cannot conclude on the effects of the intervention for children with a specific diagnosis. Parents declining participation in the patient control group explained that the requirements to their children were excessive, as there were no benefits from participating in the study (i.e., no physical activity or ambassador visits). It can be speculated that the children in the patient control group consisted of children with an interest in exercise, consequently resulting in an underestimation of the effects.

Moreover, it can be speculated that children with the most severe long-term adverse effects declined participation or dropped out of the study, limiting the generalizability and certainty of the study results. Further, the study is limited by the few completed CPET. The missing data indicate that the children with the best physical capacity completed the CPET, thus limiting the generalizability and certainty of the effects of the intervention.

## Conclusion

This study indicates that a peer-supported and supervised in-hospital physical activity intervention initiated from diagnosis may be beneficial on cardiorespiratory fitness and muscle strength in children with cancer post-treatment. The study also indicates that physical activity during treatment may improve muscle strength and physical function to a level similar to that of children without a history of cancer, although cardiorespiratory fitness requires a more targeted approach. However, the results should be interpreted with caution because of the limitations present in the study. Overall, improved physical function might not only improve the children’s long-term physical performance but may also be a core element in their social and educational rehabilitation.

## Supplementary information

ESM 1 (DOCX 15 kb)

ESM 2 (DOCX 17 kb)

## Data Availability

The datasets used during the current study are available from the corresponding author on reasonable request after the last follow-up data have been collected and published.

## Abbreviations

RESPECT: Rehabilitation including Social and Physical Activity and Education in Children and Teenagers with Cancer
LCH: Langerhans cell histiocytosis
MDS: Myelodysplastic syndrome
CPET: Cardiopulmonary exercise test
CI: Confidence interval
SD: Standard deviation
