# Supplementary material for: Children with cancer and their cardiorespiratory fitness and physical function—the long-term effects of a physical activity program during treatment: a multicenter non-randomized controlled trial
Source: J Cancer Surviv. 2023 Dec 6;19(2):672–84. doi: 10.1007/s11764-023-01499-7 (PMC11926049; doi:10.1007/s11764-023-01499-7)
Supplement: Supplementary file 1 — (DOCX 15 kb) [file 11764_2023_1499_MOESM1_ESM.docx]

Supplementary 1: Treatment protocols and modalities for the included childhood cancer survivors

| **Treatment protocols** | Intervention Group | Patient Control Group |
| --- | --- | --- |
| NOPHO ALL 2008 | 27 | 13 |
| NOPHO-DBH-AML 2012 | 8 | 3 |
| ICC APL 01 | 2 | 0 |
| Euro-LB-02 | 3 | 0 |
| Euro NET PHL-C1 interrim | 4 | 0 |
| Euro NET PHL-C2 | 1 | 4 |
| BFM NHL 2004 | 5 | 0 |
| BFM NHL 2013 | 3 | 3 |
| Euro-Ewing 99 | 5 | 2 |
| EURAMOS-1 | 4 | 1 |
| CCLG interim | 1 | 0 |
| EpSSG RMS 2005 | 3 | 2 |
| EpSSG-NRSTS 2005 | 1 | 0 |
| UKSSG | 0 | 1 |
| SIOPEL. high risk-PLADO | 1 | 0 |
| Neoadjuvant docetaxel/cisplatin/fluorouracil | 1 | 0 |
| SIOP-CNS GCT 2 | 2 | 1 |
| SIOP 2001 | 0 | 1 |
| SIOP ependynoma 2 | 1 | 0 |
| SIOP PNET 5 | 1 | 1 |
| SIOP- LGG 2004 | 0 | 1 |
| LCH-III | 1 | 0 |
| Allogeneic transplantation | 2 | 0 |
| **Treatment modalities** |  |  |
| Chemotherapy | 75 | 33 |
| Radiation therapy | 10 | 4 |
| Surgery | 19 | 9 |
| **Tumor location** |  |  |
| Central nervous system | 4 | 1 |
| Head | 2 | 1 |
| Torso | 5 | 4 |
| Upper extremity | 1 | 0 |
| Lower extremity | 8 | 3 |
