# Supplementary material for: Children with cancer and their cardiorespiratory fitness and physical function—the long-term effects of a physical activity program during treatment: a multicenter non-randomized controlled trial
Source: J Cancer Surviv. 2023 Dec 6;19(2):672–84. doi: 10.1007/s11764-023-01499-7 (PMC11926049; doi:10.1007/s11764-023-01499-7)
Supplement: Supplementary file 2 — (DOCX 17 kb) [file 11764_2023_1499_MOESM2_ESM.docx]

**Supplementary 2**: Descriptive data on cardiorespiratory fitness,

muscle strength and physical function one- year after ended treatment.

|  | n | Mean (SD) | Median [10^th^ to 90^th^ percentile] |
| --- | --- | --- | --- |
| **VO_2_peak (mL/kg/min)** |  |  |  |
| Intervention group | 52 | 37.0 ± 6.0 | 37.5 [29.1 to 45.3] |
| Patient control group | 25 | 32.3 ± 9.7 | 30.9 [22.7 to 44.5] |
| Community control group | 38 | 41.5 ± 6.6 | 40.6 [33.5 to 50.4] |
| **VO_2_peak (L/min)** |  |  |  |
| Intervention group | 52 | 1.95 ± 0.65 | 1.9 [1.2 to 3.0] |
| Patient control group | 25 | 1.83 ± 0.86 | 1.6 [0.9 to 2.9] |
| Community control group | 38 | 2.23 ± 0.66 | 2.14 [1.5 to 3.2] |
| **Max Watt (W)** |  |  |  |
| Intervention group | 52 | 148 ± 50 | 140 [90 to 210] |
| Patient control group | 25 | 139± 59 | 130 [68 to 224] |
| Community control group | 38 | 195 ± 62 | 180 [120 to 277] |
| **Sit-to-Stand (reps)** |  |  |  |
| Intervention group | 74 | 30 ± 5 | 30 [23 to 36] |
| Patient control group | 18 | 23 ± 6 | 24 [16 to 30] |
| Community control group | 90 | 29.±6 | 30 [21 to 35] |
| **Timed Up and Go** (s) |  |  |  |
| Intervention group | 75 | 3.4 ± 0.4 | 3.4 [2.9 to 4.0] |
| Patient control group | 18 | 4.3 ± 0.6 | 4.3 [3.4 to 5.1] |
| Community control group | 91 | 3.6 ± 0.6 | 3.4 [ 2.9 to 4.3] |
| **Right Handgrip Strength (kg**) |  |  |  |
| Intervention group | 75 | 27 ± 12 | 24[14to 44] |
| Patient control group | 17 | 22 ± 10 | 18.0 [12 to 37] |
| Community control group | 93 | 28 ±13 | 25 [14 to 48] |
| **Left Handgrip Strength (kg)** |  |  |  |
| Intervention group | 75 | 25 ± 12 | 22[12 to 43] |
| Patient control group | 16 | 19 ± 8 | 18 [12 to 31] |
| Community control group | 92 | 26 ± 12 | 23 [14 to 46] |
